# Postoperative delirium assessed by post anesthesia care unit staff utilizing the Nursing Delirium Screening Scale: a prospective observational study of 1000 patients in a single Swiss institution

**DOI:** 10.1186/s12871-015-0168-8

**Published:** 2015-12-18

**Authors:** A. Winter, MP. Steurer, Alexander Dullenkopf

**Affiliations:** Department of Anesthesia and Intensive Care, Kantonsspital Frauenfeld, Pfaffenholzstrasse 4, Frauenfeld, 8501 Switzerland; Department of Anesthesia and Perioperative Care, University of California, San Francisco, USA

**Keywords:** Anesthesia, Complications, Neurological, Delirium, Outcome

## Abstract

**Background:**

Delirium has become better studied, but is still only partially understood and significantly underestimated. There are some well-known risk factors, but little is known about the incidence of delirium in the diverse patient population of a post anesthesia care unit (PACU). The aim of this study was to investigate the presence of delirium using the Nursing Delirium Screening Scale (NU-DESC).

**Methods:**

1000 consecutive patients in the PACU were prospectively evaluated at the point when being ready to be transferred to the normal ward by the registered nurses of the PACU.

**Results:**

The data of 1,000 patients was recorded. 242 of the patients (24.2 %) were preoperatively classified as ASA I physical status, 664 patients (66.4 %) as ASA II. A total of 43 patients (4.3 %) presented with a delirium at the time point where they would have been transferred to the normal ward (138.4 ± 55.2 min after arrival in the PACU). 287 patients (28.7 %) of the entire group were over the age of 70 years. Considering only this subgroup, delirium was diagnosed in 30 individuals (10.5 %).

**Conclusions:**

Delirium screening with the NU-DESC, collected by nursing staff of a PACU is easily feasible and demonstrated a low incidence of delirium in the presented setting.

**Trial registration:**

German Clinical Trials Register (Deutsches Register Klinischer Studien, www.drks.de; DRKS 000005426; date of registration 4th December 2013).

## Background

In recent years, delirium occurring in hospitals has become a better studied, but still only partially understood and still significantly underestimated problem [1]. Delirium cannot only physically endanger patients, it can also be unsettling in retrospect for both patients and their relatives. Additionally, it may seriously impact short- and long-term postoperative outcomes. Examples are prolonged hospitalizations, the need for admission to a higher level of care, and even increased mortality rates [2–4]. There is also conducive evidence of negative long-term consequences, such as brain disorders (i.e. post-operative cognitive decline; POCD) [5–7].

There are some well-known risk factors for the occurrence of delirium in the hospital, such as age, neurologic comorbidity, urgent need for surgery, certain surgical procedures, etc. [8–10]. The reported incidence of postoperative delirium (POD) varies greatly, with certain patient subgroups reaching incidences of up to 80 % [11]. Much of the corresponding data comes from intensive care, geriatric, cardiac surgery or arthroplasty patients. While POD is a well-described phenomenon in patients being treated in an intensive care unit, less is known about the incidence in the more diverse patient population that frequents a post anesthesia care unit (PACU) [2, 12, 13].

There are different tests available for the detection of delirium, some of which do not require a specialization in neurology or psychiatry, but can be used bedside by trained personel, with the CAM-ICU probably representing the most frequently used in postoperative settings [3, 10, 14, 15]. Another, apparently simple instrument for early detection of delirium is the Nursing Delirium Screening Scale (NU-DESC) [16]; its score is composed of five parameters that can readily be assessed [13, 16, 17]. The Nu-DESC test was designed to be administered by a bedside nurse based on clinical observations in their routine practice, while only absorbing one minute of their time, on average. Its sensitifity is reportedly very high, with probably lower specifity [13, 15, 16]. Despite initially havin been developed for use in oncology inpatients, the NU-DESC has since been validated in a PACU under study conditions [13, 16, 17].

In this study, the presence of delirium was assessed in 1000 consecutive patients in a post anesthesia care unit of a non-tertiary hospital at the point where the patient was ready to be transferred to the surgical inpatient ward. The PACU nursing staff utilizing the NU-DESC screening tool did the assessment. This was considered to be a possible first step on the way to a broader implementation of comprehensive testing for eventually following patients with POD afterwards on the surgical inpatient ward.

The aim of this study was to get an idea of the incidence of delirium in our patient population at the time-point of transfer from PACU to ward and to test the feasibility of implementing a standard NU-DESC screening by PACU nursing.

## Methods

The study was conducted after approval by the local ethics committee (Kantonale Ethikkomission Thurgau, Switzerland; protocol number 01.53.01; Chairperson Dr. R. Andenmatten; approved 26^th^ March 2013) and the registration with the German Clinical Trials Register (Deutsches Register Klinischer Studien, www.drks.de; DRKS 000005426). The requirement for written informed consent was waived by the ethics committee.

During approximately 6 months, starting in May 2013, 1000 consecutive patients in the post anesthesia care unit of the Kantonsspital Frauenfeld (www.stgag.ch) were prospectively evaluated for the presence of delirium utilizing the Nursing Delirium Scale. Patients were assessed at the end of their PACU stay when deemed ready to be transferred to the surgical inpatient ward. All patients admitted to the PACU during this time period were included, except for patients who did not speak German well enough to be assessed by the nursing staff of the PACU.

A team of eight registered nurses of the PACU were trained on both the NU-DESC and the clinical significance of the delirium before they performed the delirium screening on the patients. Training consisted of two lectures, followed by discussion and was conducted by one of the authors (AD). During the study period, PACU visits were done repeatedly by two of the authors (AW and AD) to assist in questions around the delirium assessments.

In addition to the NU-DESC score we also recorded the length of stay in the PACU, age, gender, surgical specialty/ procedure and the type and duration of the anesthetic.

The nursing staff of the post anesthesia care unit recorded the NU-DESC. In order to do so, five parameters were captured; each with a sub score of 0–2 points (Table 1). A total of ≥ 2 points indicated the presence of a delirium [16].

**Table 1:** Nursing delirium screening scale [16]

Symptom	Symptom rating
1 Disorientation	
Verbal or behavioral manifestation of not being oriented to time or place or misperceiving persons in the environment	
2 Inappropriate behavior	
Behavior inappropriate to place and/or for the person; e.g., pulling at tubes or dressings, attempting to get out of bed when that is contraindicated, and the like.	
3 Inappropriate communication	
Communication inappropriate to place and/or for the person; e.g., in-coherence, non-communicativeness, nonsensical or unintelligible speech.	
4 Illusions/ Hallucinations	
Seeing or hearing things that are not there; distortions of visual objects.	
5 Psychomotor retardation	
Delayed responsiveness, few or no spontaneous actions/words; e.g., when the patient is prodded, reaction is deferred and/or the patient is unarousable.	

Annually, roughly 8400 anesthetics are performed at the Kantonsspital Frauenfeld, serving the surgical specialties of orthopedics, general surgery, vascular surgery, hand surgery, plastic surgery, gynecology, urology, and ENT.

The PACU is available to inpatients over the age of 16 years on weekdays from 09:00 to 20:00. Due to its limited capacity, it is used to recover roughly 20 patients per day. The remaining inpatients are directly transferred to the surgical inpatient ward; ambulatory patients are cared for in a separate outpatient clinic in the postoperative phase, obstetric patients return to the labor and delivery ward after their surgical procedure. In addition, any patient requiring either prolonged intubation and ventilation or vasopressor therapy will be transferred to the intensive care unit postoperatively. The anesthetist in charge ultimately makes the triage decision for where a given patient will be recovered.

During the entire year of 2013, a total of 3078 patients were cared for in the PACU.

The readiness for transfer of a patient to the surgical inpatient ward is determined by the nursing staff of the PACU on the basis of predefined criteria (Table 2).

**Table 2:** Criteria to determine patient readiness for transfer from the post anesthesia care unit to the surgical inpatient ward

• Ability to maintain and protect airway	
• Normalization of respiratory status	
• Cardiovascular stability without medical support	
• No significant hemorrhage	
• Sufficient diuresis	
• Adequate pain and nausea control	
• Body temperature within normal limits	
•After spinal anesthesia: sensory level below thoracic 10	

### Conduct of anesthetic

The standard guideline for general anesthetics at our institution was applied to this patient population. However, no specific data on adherence and variance according to the anesthesiologist in charge were collected. Our guideline encompasses a fasting period of two hours for clear liquids and six hours for solid food, a pharmaceutical premedication with Midazolam 7.5 mg PO in patients ≤ 70 years of age, reducing the dose to 3.75 mg PO in patients older than 70 years and entirely omitting premedication in patients over 80 years old.

Both the induction and maintenance of the anesthetic are done using Propofol. Sevoflurane is used for maintenance in rare instances. Fentanyl and Remifentanil are used for intra-operative analgesia, supplemented with 0.1 mg/kg Morphine IV, Paracetamol and/or Metamizol and/or NSAIDs IV for post-operative pain therapy. Most anesthetics are guided by Bispectral Index (BIS) monitoring.

### Statistics

The aforementioned demographic and procedure relevant data is presented descriptively. The group of patients that presented with delirium was compared with the group of patients without delirium (*t*-test for independent samples or chi-square test for qualitative data, *p* < 0.05 was used as significance cut off).

## Results

The data of 1,000 patients was recorded. The surgical subspecialties that were represented most frequently were general surgery and orthopedics, each with 302 patients (30.2 %), followed by gynecology 183 (18.3 %), and urology 104 (10.4 %).

242 of the patients (24.2 %) were preoperatively classified as ASA I, 664 patients (66.4 %) as ASA II, 83 patients (8.3 %) as ASA III, and 10 patients (1.0 %) as ASA IV.

Table 3 shows the results of the NU-DESC assessment of all patients. A total of 43 patients (4.3 %) presented with a delirium at the time point where they would have been transferred to the surgical inpatient ward (138.4 ± 55.2 min after arrival in the recovery room).

**Table 3:** NU-DESC score result of all patients

Nu-DESC (0 – 10)	n	%
0	928	92.8
1	29	2.9
2	17	1.7
3	8	0.8
4	8	0.8
5	1	0.1
6	3	0.3
7	2	0.2
8	2	0.2
9	0	0
10	2	0.2

NU-DESC = Nursing Delirium Screening Scale

The demographic data for all patients and the comparison between the group of patients with delirium, as well as the patients without delirium is shown in Table 4, and Table 5 respectively.

**Table 4:** Demographic and surgery-related data for all patients

Parameter		Mean or n	± SD or %
Sex	M / F	598/402	59.8/40.2 %
Age	Years	58.0	±18.3
Duration of surgery	min	92.5	±60.3
Anesthesia duration	min	166.1	±71.2
Type of anesthesia	GA / RA	929/71	92.7/7.1 %
Stay duration in PACU	min	138.4	±55.2
By Surgical Specialty		n	%
General Surgery		302	30.2
Orthopedics		302	30.2
Gynecology		183	18.3
Urology		104	10.4
ENT surgery		64	6.4
Plastic Surgery		36	3.6

*n* = number, SD = standard deviation; f = female; m = male; AA = general anesthesia; RA = regional anesthesia; PACU = post anesthesia care unit

**Table 5:** Patients with delirium (NU-DESC score ≥ 2) compared to patients without delirium

		Patients without delirium		Patients with delirium		Comparison
Parameter	Unit	mean	± SD	mean	± SD	*
Sex	f / m	574/383	60.0/40.0 %	24/19	55.8/44.2 %	0.59
Age	Years	57.3	±18.1	72.9	±16.1	<0.0001*
Duration of surgery	min	92.9	±60.3	85.6	±59.0	0.45
Duration of anesthesia	min	166	±71.3	167.5	±70.5	0.90
Type of anesthesia	GA / RA	960/40	96.0/4.0	43/0	100/0 %	0.17
Stay duration in PACU	min	138.9	±55.5	128.6	±48.2	0.24
By Surgical Specialty		n=	%	n=	%	
General Surgery		291	30.4	11	25.5	
Orthopedics		288	30.1	14	32.6	
Gynecology		177	18.5	6	14.0	
Urology		98	10.2	6	14.0	
ENT surgery		63	6.6	1	2.3	
Plastic Surgery		36	3.8	0	0	

*n* = number, SD = standard deviation; f = female; m = male; GA = general anesthesia; RA = regional anesthesia; PACU = post anesthesia care unit; * *P* <0.05 = statistically significant result (*t*-test or chi-square test, as appropriate)

287 patients (28.7 %) of the entire group were over the age of 70 years. Considering only this subgroup, delirium was diagnosed in 30 individuals (10.5 %).

Also, the surgical subspecialties of orthopedics (4.6 %) and urology (5.8 %) conveyed a disproportionately higher number of patients with delirium. With an age of 62.4 ± 17.5 years, and 65.5 ± 16.0 years, respectively, those patients were slightly older on average than the entire group (58.0 ± 18.3 years, *p* <0.05).

In the group of orthopedic patients with an age of 70 years or older (*n* = 111) 12 patients were diagnosed with delirium (10.8 %), in the corresponding urological sub-group (*n* = 43), there were 5 patients (11.6 %).

## Discussion

In this study, the presence of delirium in patients who were recovered in a PACU after a non-cardiac surgical intervention was prospectively studied. The instrument utilized for the detection of delirium was the Nursing Delirium Screening Scale (NU-DESC; with a delirium defined as a score of ≥ 2 points). NU-DESC was conducted by the nursing staff in the PACU prior to the transfer of the patient to the surgical inpatient ward.

The most prominent result of the study was that delirium assessment using the NU-DESC test was very easily feasible in this setting. There was a relatively low incidence of delirium with only 4.3 %, compared to some previously cited rates [2, 13, 17]. Even when just considering the at-risk subgroups, the rates of delirium remain low. There are a number of possible reasons for these findings, which are discussed below.

The delirium that occurs in the hospital is still a poorly understood phenomenon. In recent years, however, awareness of the problem has increased and the research surrounding delirium was intensified [2, 3, 10, 12]. As a consequence, many risk factors that can lead to delirium have recently become known [7, 9, 10, 18]. Elderly patients undergoing major surgery are particularly at risk [7, 9, 10, 18, 19]. It also appears justified to distinguish between cardiac surgery and non-cardiac surgery, since the former is associated with a greater risk for both the incidence of delirium as well as the development of long term brain dysfunctions [1, 10, 20, 21]. Unfortunately, delirium is not always a reversible condition. It can be associated with serious long-term consequences, which affect not only cognitive performance, but also may result in prolonged stay in the hospital and intensive care unit, higher costs, and even increased rates of complications and mortality [1, 4, 5, 21–25].

In our study, we found a relatively low incidence of delirium. The most obvious reason for that seems to stem from our patient population. The incidence of delirium can reach rather high incidences in specific sub-groups. In certain patient populations in intensive care units it can reach levels of up to 80 % [26]. Elderly orthopedic trauma patients have been reported to have a frequency of delirium of almost 50 % in the PACU [19, 27]. In contrast to these sub-groups of at-risk patients, our patient population was rather young and healthy, as is reflected in their low ASA physical status scores. Radtke and colleagues [13] reported an incidence of 25 % for delirium in their recovery room using the NU-DESC. In their series, only 75 % of all patients had an ASA physical status of either I or II. In contrast, our series included 90 % of patients with an ASA physical status of I or II, likely reflecting the difference in patient population between a tertiary care hospital (Charité, Berlin, Germany) and our institution. Interestingly, in a recent study by Card [12] et al. a very similar rate of delirium to our study was reported using the CAM-ICU test, which may be more specific but less sensitive than the NU-DESC in a PACU [15].

Another difference between our work and the mentioned studies using the NU-DESC score lies in the fact that in our study the assessment was done by regular nursing staff of the post anesthesia care unit rather than specifically trained psychologists, psychiatrists, or study nurses [13]. Especially item 5 of the NU-DESC data collection sheet (Table 1) asks for a comparison between the given patient and a reasonable, adequately acting normal person. PACU nursing staff may be more likely to gauge someone who is not yet fully oriented or still slightly sedated postoperatively as normal, than an independent investigator who is not familiar with this postoperative setting. Accordingly Haenggi and colleagues noted that the exact assessment and documentation of the patient’s sedation scale should be a key component when studying postoperative delirium [11]. In a study with patients in an intensive care unit, 53 % of the patients were found to be delirious when screened only with the CAM-ICU score. This number was significantly reduced to 31 % when the more sedated patients (Richmond Agitation Sedation Scale, RASS score of −2 or −3) were excluded [11].

Even if not quantified systematically, the acceptance of the additional task of delirium screening by the team staff was high, and with the test being in fact performed in about one minute, on average, the additional burden was not a topic.

The overall utility of the NU-DESC can also be questioned. There is the notion that early detection of delirium could lead to more effective treatment and the reduction of the negative consequences,[14, 28, 29] despite there being no uniform consensus on the exact treatment modalities [2]. The detection of delirium remains challenging because of the variety of manifestations: a clear confusional state with agitation on one end of the spectrum, and a hypoactive state on the other [12]. Accordingly, the diagnosis should not be supported on an individual estimation, but must be assessed using an appropriate assay [13, 17, 26, 28, 30]. The NU-DESC has been validated against more extensive tests and impresses mainly with its simple implementation, making it well suited for routine use in a PACU [13, 16]. Applying the NU-DESC to a given patient requires not more than 1 min [16]. However, there are some inconsistent data reported about sensitivity and specificity of the test [13, 17]. A recent review of different delirium bedside screening tools characterized the NU-DESC as highly sensitive (>95 %) but considerably less specific (>70 %) [15]. Taking this into account, it is difficult to predict in what manner using the NU-DESC test could induce bias regarding the incidence of delirium, when compared to other tests.

It would be tempting, yet unrealistic to assume that our anesthetic management contributed to the relatively low incidence of delirium in our patient population. However, in contrast to our standard, i.e. maintaining anesthesia using Propofol, Radtke and colleagues [13] chose volatile anesthetics for over half of their patients, and a study by Neufeld and colleagues [17] used volatile anesthetics in over 90 %,. Since the anesthetic management for our patients did not follow a given study protocol, we abstain from further speculation and a more detailed analysis at this point. The same limitation stands true for the role of pharmacological premedication with benzodiazepines given to our patients prior to anesthesia, which was not controlled as would have been under study conditions.

In addition, the duration of stay in the PACU and thus the time between the end of anesthesia and the assessment of the NU-DESC score may play a roll. This time was almost twice as long in our study, compared to that of Neufeld et al. who did not assess their patients prior to transfer to the surgical inpatient ward; they rather did so when reaching an Aldrete score of 9 points, which was the case after about 45 min [17]. When considering the initial recovery period after general anesthesia as a form of delirium, it is not surprising that the incidence of delirium decreases with increasing time after the anesthesia [11].

The NU-DESC score is potentially too simple to be used as a widespread screening tool in post anesthesia care units, especially when dealing with patients that are slow in their reactions. The clinical benefit of the NU-DESC could be increased by virtue of more intense training of the PACU nurses in order to achieve a more uniform assessment. It has been described for intensive care units, that regular feedback on the incidence of delirium, completeness of patient evaluations, and occasional reference comparison to the assessment of an uninvolved psychiatrist helped the successful implementation of the screening for delirium [28, 31]. The same approach could be supportive for PACUs, but more studies in the immediate postoperative setting are warranted. At the same time a longitudinal extension of the study that would investigate the course of patients with delirium after their transfer to the surgical inpatient ward could contribute further valuable insight into the disease. In fact, the relevance of PACU delirium screening will have to be examined in the context of its actual association with adverse outcomes in future trials.

A limitation to our study is that the timing of the delirium screening is somewhat vague. Choosing fixed time intervals from admission to the PACU, and probably adding repeated assessments would have made our data more objective and reproducible. However, our study was thought to be only a first step in a broad implementation of delirium screening in postoperative patients. Hence, the feasibility of the test and estimating the proportion of patients with a need for follow-up was our first priority.

We did not attempt to verify known predictors for developing delirium postoperatively. It also must be mentioned that the comparison of patients with delirium and the patients without delirium lacks an appropriate power analysis.

## Conclusions

In summary, delirium screening with the NU-DESC, collected by nursing staff of a PACU is easily feasible but may result in a rather low incidence of delirium. Amongst other reasons, mainly the diversity of patient populations, interference of postoperative sedation and the time of assessment could be the reasons for that.
